# Multilevel calcium pyrophosphate dihydrate deposition in cervical ligamentum flavum: clinical characteristics and imaging features

**DOI:** 10.1186/s12891-021-04812-6

**Published:** 2021-11-04

**Authors:** Yueh-Hsiu Lu, Hsi-Hsien Lin, Hsuan-Ying Chen, Po-Hsin Chou, Shih-Tien Wang, Chien-Lin Liu, Ming-Chau Chang

**Affiliations:** 1 Department of Orthopedics and Traumatology, Taipei Veterans General Hospital, No. 201, Sec. 2, Shipai Rd., Beitou District, Taipei, 11217 Taiwan Republic of China; 2 Department of Orthopedics, Changhua Christian Hospital, No. 135, Nanxiao St., Changhua County, Changhua, 500054 Taiwan Republic of China; 3 Institute of Biomedical Sciences, College of Life Sciences, National Chung Hsing University, No. 145 Xingda Rd., South District, Taichung, 40227 Taiwan Republic of China; 4 School of Medicine, National Yang-Ming University, No.155, Sec. 2, Linong St., Beitou Dist, Taipei City, 11217 Taiwan Republic of China; 5 Orthopedics & Sports Medicine Laboratory, Changhua Christian Hospital, No. 235 Shi-Guan Rd., Changhua County, Changhua, 50006 Taiwan Republic of China

**Keywords:** Calcium pyrophosphate dihydrate, Cervical ligamentum flavum, Magnetic resonance imaging, Ossification of ligamentum flavum

## Abstract

**Background:**

Involvement in cervical ligamentum flavum is a rare manifestation of the calcium pyrophosphate dihydrate deposition disease. Only few cases of this condition have been reported. We revealed eighteen cases of CPPD in cervical ligamentum flavum that diagnosed at a single medical center. In our case series, clinical characteristics and magnetic resonance imaging findings of patients are described.

**Methods:**

We retrospectively reviewed the medical charts and imaging studies of the eighteen patients with pseudogout attack of the cervical ligamentum flavum. In addition, we discussed the differences between this disease and ossification of ligamentum flavum in image manifestations.

**Results:**

There were fourteen men and four women aged between 59 and 87 years. Diabetes mellitus and hypertension were the most common comorbidities. Myelopathy and neck pain were presented in most patients. C4–5 and C5–6 were attacked most frequently, and multiple- rather than single-level involvement could be observed in our series. “Acute on chronic phenomenon” was a specific magnetic resonance image finding in patients whose symptom durations were between 2 to 5 months. Compared to ossification of ligamentum flavum, calcium pyrophosphate dihydrate crystal deposition had different image signs, including morphology, side of the involved ligament, no continuity with the lamina, acute on chronic phenomenon, and presence of retro-odontoid mass.

**Conclusions:**

Nodular calcifications in cervical ligamentum flavum raise highly suspicion for calcium pyrophosphate dihydrate deposition and must be diagnosed by histological examination and polarized light microscopy. This disease is different from ossification of ligamentum flavum, and it could be recognized by specific image features.

## Introduction

Calcium pyrophosphate dihydrate (CPPD) crystal deposition disease, also known as pseudogout, was first described by Zinan and Sitaj in 1958 [1]. It is thought to be a disease of aging and patients younger than 50 years of age are less commonly affected [2]. The pathogenesis of CPPD deposition disease is still unknown, but the disease can be associated with some metabolic or endocrine diseases, including diabetes mellitus, hypertension, hyperparathyroidism, hypothyroidism, hypophosphatasia, hemocromatosis, gout, and rheumatoid arthritis [3]. Idiopathic CPPD deposition disease is the most common form. CPPD crystals deposit commonly at joints of extremities and sometimes at the thoracolumbar spine. However, the cases with CPPD in cervical spine is a rare entity, and patients were primarily found in Japan and North Africa [4]. The attack of crystals in intervertebral discs, articular cartilages, joint capsules, and ligaments could result in spinal lesions. However, most patients with calcifications of the intervertebral discs and articular cartilages are asymptomatic [5]. Conventional radiography and sonography are main imaging techniques for the diagnosis of peripheral CPPD and the computed tomography is commonly used to detect axial CPPD [6, 7]. In the presented case series, we report 18 patients with calcification of cervical ligamentum flavum who required surgical treatment and finally were confirmed to suffer from CPPD deposition disease by pathologic diagnosis. Compared with previous reports which discussed the findings of pathophysiology, laboratory tests, clinical manifestation, computed tomography (CT) scan, and various pathological studies, our report showed magnetic resonance imaging (MRI) findings and other clinical characteristics. In addition, CPPD deposition disease in the ligamentum flavum is frequently mistaken for ossification of ligamentum flavum which is commonly found in the lower thoracic spine and possibly identified in the cervical spine [8]. To date, there is no study to examine the difference in imaging features between the two diseases. Therefore, we also describe the comparison of imaging findings in this study.

## Materials and methods

From January 2014 to January 2020, 18 patients with CPPD deposition in cervical ligamentum flavum were retrospectively analyzed and formed the basis of this study. All these cases received surgical treatment and were diagnosed by histological examination at our hospital, a teaching hospital which provides tertiary patient care. In these subjects, only two patients had a previous diagnosis of CPPD and the knee joint was involved. In all the other patients, cervical spine involvement was the first presentation of the disease. Table 1 showed their basic clinical data, including age, gender, comorbidities, symptoms, symptom duration, and types of surgery. The data of blood examinations were reviewed if data was available. Anteroposterior and lateral radiographs were obtained in all patients. Computed tomography (CT) scans were available in five patients. MRIs (1.5-T machine, Magnetom Vision, Siemens Medical Systems, Iselin, NJ; Signa, GE Medical Systems, Milwaukee, WI) were performed in all patients preoperatively to evaluate spinal cord compression and confirm abnormal soft tissue structure. We recorded the signal intensity of the lesions in the ligamentum flavum on T1- and T2- weighted images as low, intermediated, high-signal intensity, as compared with that of the near-by bone, disc, or cerebrospinal fluid. Mixed signal intensity meant that the lesion showed both hyperintensity and hypointensity in the ligamentum flavum. The morphology, affected levels and presence of retro-odontoid mass were also recorded. The patients included in this study were treated initially by pharmacological treatments, such as acetaminophen and non-steroidal anti-inflammatory drugs. All patients received surgical treatment due to failed conservative treatment. The surgical options were consisted of posterior decompressive surgery alone and posterior decompression with posterolateral fusion. In addition, calcified lesions in ligamentum flavum were removed entirely and every surgical specimen was checked by histologic examination and polarized light microscopy. Postoperative results were evaluated according to Frankel scale [9].

**Table 1:** Basic clinical data

Case	Age/Sex	Comorbidities	Symptoms	Duration	Type of surgery
1	70/M	DM	Neck pain Myelopathy	2 months	Decompression with fusion
2	85/M	Absent	Myeloradiculopathy	10 months	Decompression
3	59/M	HTN, Arrhythmia	Myeloradiculopathy	3 months	Decompression
4	80/M	DM, HTN	Myelopathy	4 moths	Decompression with fusion
5	66/M	HTN, hyperuricemia	Neck pain Myeloradiculopathy	10 months	Decompression with fusion
6	72/M	DM, HTN	Neck pain Radiculopathy	3 months	Decompression
7	86/M	HTN, COPD	Neck pain Myeloradiculopathy	18 months	Decompression
8	79/M	Absent	Myelopathy	2 months	Decompression
9	87/M	HTN, COPD	Myeloradiculopathy	2 months	Decompression
10	73/M	DM, HTN	Neck pain Myeloradiculopathy	4 months	Decompression
11	78/F	HTN CAD	Myelopathy	18 months	Decompression
12	70/F	DM, CAD	Neck pain Myeloradiculopathy	24 months	Decompression
13	77/M	HTN, ESRD	Neck pain Myeloradiculopathy	24 months	Decompression
14	81/M	HTN	Neck pain Myelopathy	9 months	Decompression
15	67/F	ESRD	Neck pain Myelopathy	15 months	Decompression with fusion
16	81/M	DM	Myeloradiculopathy	9 months	Decompression
17	78/F	DM, CAD	Neck pain Myeloradiculopathy	5 months	Decompression with fusion
18	77/M	HTN	Neck pain Radiculopathy	12 months	Decompression

*M* Male, *F* Female, *DM* Diabetes mellitus, *HTN* Hypertension, *COPD* Chronic obstructive pulmonary disease, *CAD* Coronary arterial disease, *ESRD* End stage renal disease

## Results

**Fig. 1:**
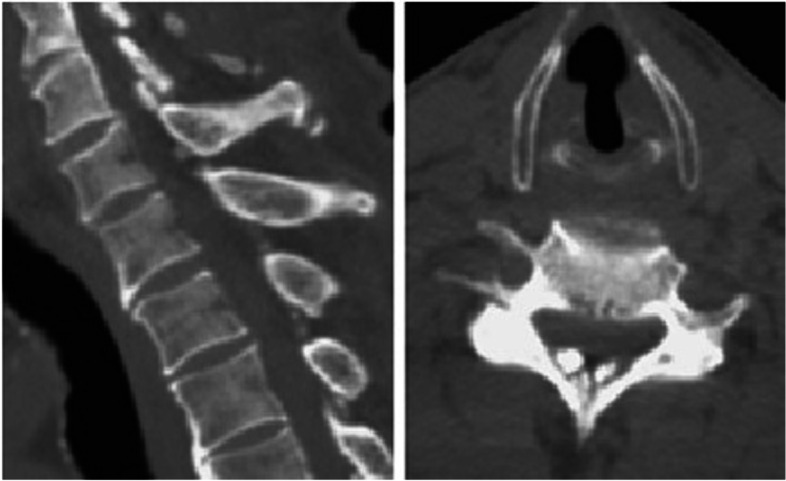
Cervical computed tomography of case 5. Compression of the spinal cord by two main calcified masses was observed on sagittal and axial sections. There is no calcification or ossification of the posterior longitudinal ligament

The series included fourteen males and four females (cases 11, 12, 15, and 17), and the mean age was 75.9 years (range: 59 to 87 years). The mean age of the women was 73.2 years (range, 67 to 78 years), while the mean age of the men was 76.6 years (range, 59 to 87 years). The mean symptom duration was 9.7 months (range: 2 to 24 months). Comorbidities were present in sixteen patients and consisted of hypertension (*n* = 10), diabetes mellitus (*n* = 7), coronary artery disease (*n* = 3), end stage renal disease (*n* = 2), chronic obstructive pulmonary disease (n = 2), arrhythmia (n = 1), and hyperuricemia (n = 1). Besides, the patients presented with myelopathy (*n* = 6), radiculopathy (n = 2), myeloradiculopathy (*n* = 10) and neck pain (*n* = 11). In available data of blood examinations, there was no significant difference in the level of serum uric acid and electrolytes. None of the eighteen patients did the plain radiographs of the cervical spine that contributed to the final diagnosis. CT scans that performed in five patients (cases 1, 5, 7, 13, and 18) revealed oval-shaped calcifications located ventrally to the lamina (Fig. 1). These lesions had clear margins and had no connection with the adjacent lamina. They were separated by ligamentum flavum which showed relative low density compared with that of calcified lesions and lamina in the CT images. In addition, the lesions presented bilaterally and paramedially along the midline without extending to posterior facet joint.

**Fig. 2:**
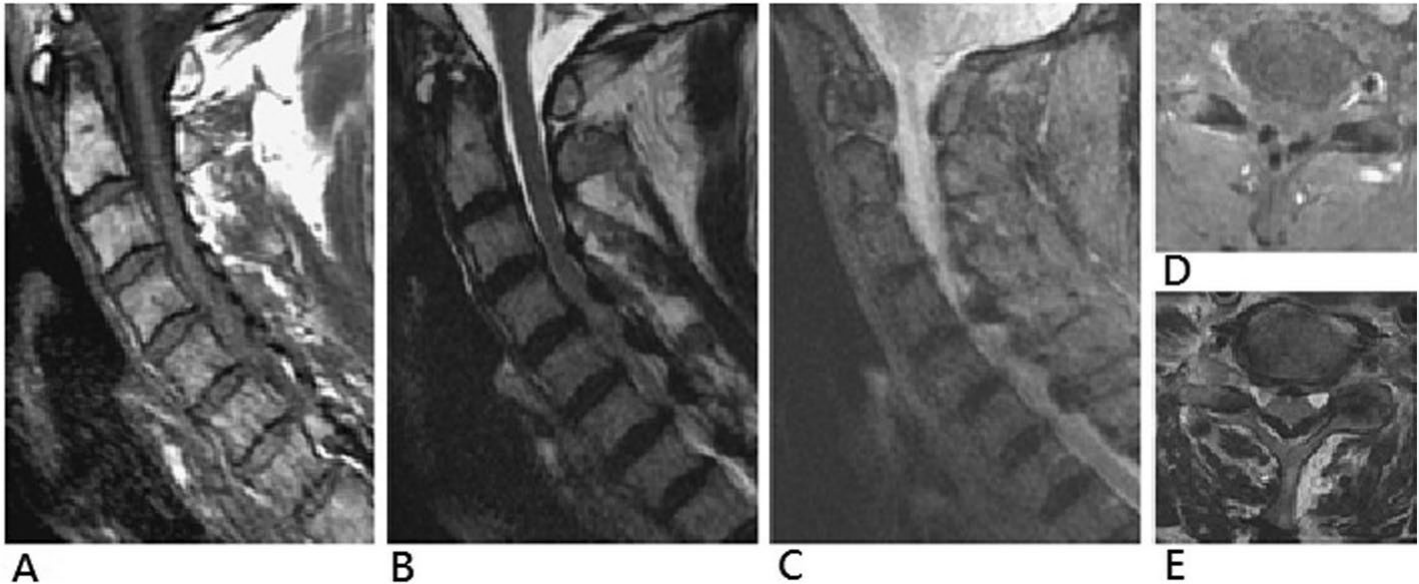
Magnetic resonance imaging of case 1. Nodular masses with low-signal intensity in both T1-weighted imaging (**A** and **D**) and T2-weighted imaging (**B** and **E**) were observed at the interlaminar spce of C4–5, C5–6, and C6–7. These lesions were surrounded by areas of intermediate-sginal intensity area on T1-weighted imaging and by areas of high-signal intensity area in T2-weighted imaging, and the short tau inversion recovery (STIR) sequence (**C**) was used to demonstrate the edematous change

**Fig. 3:**
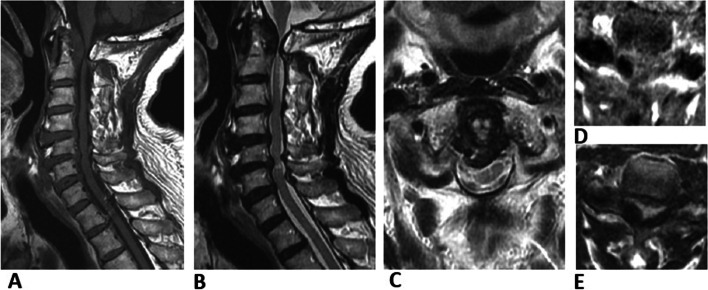
Magnetic resonance imaging of case 15. Nodular masses with low-signal intensity in both T1-weighted imaging (**A** and **D**) and T2-weighted imaging (**B** and **E**) are seen at the interlaminar spce of C5–6 and C6–7. Magnetic resonance imaging demonstrated a retro-odontoid mass severely compressing the spinal cord (**C**)

MRIs were performed in all patients and the results were listed in Table 2. Nodular lesions were presented in all cases. The mid-cervical spine was often affected, especially at C4–5 (9 in 18 patients, 50%) and C5–6 (10 in 18 patients, 56%) level. C1–2 and C7-T1 were least affected level. Both were just found once among patients. Multilevel involvement was more common than single level involvement, fifteen patients (83%) and three patients (17%) respectively. Furthermore, nine patients (50%) had two levels of involvement, four patients (22%) had three levels of involvement, and two patients (11%) had four levels of involvement. In eight patients (cases 1, 3, 4, 6, 8, 9, 10, and 17), the nodular lesion showed homogenous low-signal intensity and the signal was even lower than cerebrospinal fluid in T1-weighted image. Surrounding each nodular lesion, intermediated-signal change was noted (Fig. 2A, D, Fig. 3 A, and D). In T2-weighted image, the nodular lesions still showed homogenous low-signal intensity, surrounded by intermediated- to high-signal intensity (Fig. 2B, E, Fig. 3 B, and E). The low-signal intensity on both T1- and T2-weighted image referred to a calcified lesion. Around the lesions, the intermediated-signal change on T1-weighted and intermediated- to high-signal change referred to the edematous reaction. We described this condition as “acute on chronic phenomenon” and it might present an acute or subacute inflammatory reaction. The further short tau inversion recovery (STIR) sequence in MRI was used to demonstrate this edematous change (Fig. 2C). In these eight patients with “acute on chronic phenomenon”, their mean duration of symptoms was 3.1 months (range: 2 to 5 months) (Table 1). For patients whose duration of symptoms were longer (10 patients, mean duration: 14.9 months, range: 9 to 24 months), lesions just showed homogenous low-signal intensity on both T1- and T2-weighted images without “acute on chronic phenomenon”. Retro-odontoid lesions were found on thirteen patients (72%). Five patients (case 2, 4, 10, 11, and 13) had no obvious calcification surrounding the peri-odontoid process.

**Table 2:** MRIs findings of the lesions in cervical ligamentum flavum

Case	Morphology	Affected levels	Acute on chronic phenomenon	Retro-odontoid mass
1	Nodular	C3–4, C4–5, C5–6, C6–7	Present	Present
2	Nodular	C5–6, C6–7	Absent	Absent
3	Nodular	C5–6	Present	Present
4	Nodular	C3–4, C7-T1	Present	Absent
5	Nodular	C3–4, C4–5	Absent	Present
6	Nodular	C2–3, C6–7	Present	Present
7	Nodular	C1–2, C2–3, C3–4	Absent	Present
8	Nodular	C2–3, C3–4, C4–5	Present	Present
9	Nodular	C3–4, C4–5	Present	Present
10	Nodular	C3–4, C4–5, C5–6, C6–7	Present	Absent
11	Nodular	C4–5, C5–6	Absent	Absent
12	Nodular	C4–5, C5–6, C6–7	Absent	Present
13	Nodular	C2–3	Absent	Absent
14	Nodular	C2–3, C3–4	Absent	Present
15	Nodular	C5–6, C6–7	Absent	Present
16	Nodular	C4–5, C5–6, C6–7	Absent	Present
17	Nodular	C4–5, C5–6	Present	Present
18	Nodular	C5–6	Absent	Present

**Fig. 4:**
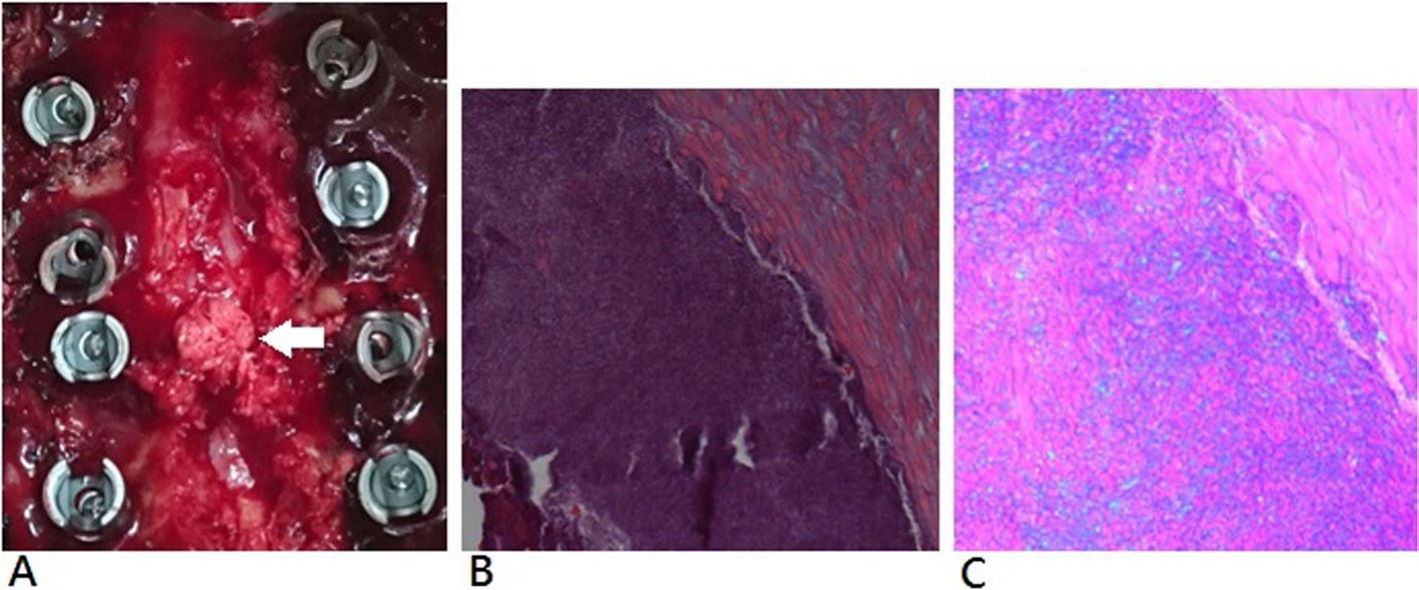
Intraoperative photograph and pathological findings. **A** The nodular lesion with fine granules and chalky white in color (white arrow). **B** When stained with hematoxylin and eosin stain, the lesion shows deeply blue, basophilic appearance (Original magnification × 200). **C** Rod-shaped crystals with positive birefringence were found under polarized light microscopy (Original magnification × 400)

Thirteen patients received posterior decompressive laminectomy alone and five patients (cases 1, 4, 5, 15, and 17) received posterior laminectomy with posterolateral fusion and instrumentation by lateral mass screws. The gross appearance of the calcified ligamentum flavum from a patient who underwent laminectomy with posterolateral fusion was shown in Fig. 4A. Abundant calcified deposits within ligamentum flavum were observed intraoperatively and did not adhere to the dura mater. Nevertheless, these crystals lead to spinal cord compression. The nodule was composed of fine granules and chalky white in color. Histopathologic examination of the specimen revealed a prominent hyaline degeneration and calcification in the fragment of ligamentum flavum. Endothelial cell, fibroblast, and giant cells were observed in the granulation tissue. There was mild inflammatory change in the selective cases, including cases 1, 3, 4, 6, 8, 9, 10, and 17. No osteoid proliferation or bone formation could be observed. The hematoxylin and eosin stain revealed a basophilic appearance (Fig. 4B). CPPD deposition with rod-shaped crystals was demonstrated under the polarized microscope (Fig. 4C). In this study, there was no specific surgical complication. In eight patients whose duration of symptoms was less than 5 months, they were discharged with two to three grades of neurologic improvement according to Frankel scale. In cases of 12 and 13 with 24 months of symptoms duration, they had just one grade of improvement. Other patients had two grades of neurologic improvement.

## Discussion

CPPD deposition in the ligamentum flavum at the cervical spine is extremely rare. According to the English-language literatures, most of case series about the disease were from Japan [10–13]. Nevertheless, the prevalence of this condition in Blacks is still under-recognized. Two patients were confirmed as the diagnosis of CPPD deposition in six Black cases with calcification of cervical ligamentum flavum in the Caribbean island [14]. In this study, we identified 18 cases at a single medical center in recent 8 years, raising the possibility that the incidence of this condition could be definitely underestimated in East Asia. Similar to CPPD deposition in cervical ligamentum flavum, ossification of the ligamentum flavum (OLF) in the cervical spine is also rare and it is generally understood that they are different diseases histologically.

Kenneth H. Fye [3] declared that CPPD deposition in cervical spine was a disorder of elderly women with tendency of calcium deposition. In their report, 80% of patients were women, but our series showed a predominance in men (14 in 18 patients, 78%). This could be explained by the fact that the mean age of male population is older than that of female population (76.6 and 73.2 years, respectively). Moreover, Miyazawa et al. [15] reported the mean age of the presented patients with OLF in the cervical spine was 56.3 years. However, our study found a mean age of 76 years, suggesting that CPPD deposition in the cervical spine has a later onset than that of the OLF in the cervical spine. In addition to aging and gender, metabolic disorders have been shown to be associated with calcium deposition as well. Diabetes mellitus (incidence from 40 to 60%), hypertension (incidence from 25 to 40%), and atherosclerosis (incidence from 35 to 50%) were most frequently encountered [16] and these comorbidities were consistent with our findings. In various symptoms of CPPD deposition in the cervical spine, cervical myelopathy was the most prevalent one, and it frequently resulted in paresthesia in the upper limbs, deterioration in fine motor skills, and difficulty in walking. A noticeable finding in our case series was that neck pain is a common complaint among patients (11 of 18 patients). Literature has shown that acute neck pain could be caused by CPPD crystal-induced arthritis of the lateral atlantoaxial joint [17]. In addition, Kobayashi et al. [18] reported a case with acute neck pain that resulted from CPPD attack of the cervical ligamentum flavum without facet arthritis. Although we can not conclude that neck pain in our case series is due to CPPD deposition in ligamentum flavum, acute neck pain should be considered a symptom for the diagnosis of CPPD deposition disease.

Markiewitz et al. [19] displayed that 33.8% of patients with CPPD deposition disease had axial involvement, mostly affecting the lumbosacral spine (87%). The cervical spine was less commonly involved. The midcervical spine was most frequently attacked among cervical spine. Hisatoshi et al. [11] reported a case series with 8 patients suffered from CPPD deposition disease, and 81% of the patients’ lesions were located between C4–5 and C6–7 levels. This finding was compatible with our data that the most commonly affected levels were C4–5 and C5–6. C5 segment has the greatest dynamic stress in the cervical spine and the elastic tissue is prone to undergo degeneration [20]. The ligamentum flavum is thinner in the cervical region than that in the thoracic or lumbar region. However, the elastic tissue is more abundant in the cervical spine. Yoshida et al. [21] and S-H Lin et al. [22] reported three cases with CPPD deposition in multilevel of ligamentum flavum. However, most patients (83%, 15 in 18 patients) had at least involvement of two intervertebral levels in our series. We believed that there is no tendency for CPPD deposition to affect just single level. Furthermore, OLF was commonly identified in the thoracic spine or the thoracolumbar junction, and cervical spine was a rare target for OLF [23]. However, when OLF occurs in the cervical spine, there is no common location and single-level involvement is predominant [15].

In order to clearly detect the cervical myelopathy, spinal cord compression, other soft-tissue changes and fluid effusion, we performed MRI for all patients. Sushil et al. [24] reported three diagnostic features of CPPD deposition disease in MR images, including: (1) mostly isointense lesion in the T1-weighted image, (2) a mixed density mass in T2-weighted image, and (3) a peripheral enhancement on the postcontrast MRI images. In term of morphology in sagittal imaging, all lesions had the nodular-type appearance, rather than beat-like or mound-like appearance that commonly observed in cases of ossification [25]. Besides, in T1-weighted image, the nodular lesion showed low-signal intensity, and the signal intensity was even lower than that of the cerebrospinal fluid. In T2-weighted image, the nodular lesion showed low-signal intensity as well. In addition, these lesions were surrounded by areas of intermediate signal intensity in T1-weighted imaging and by areas of high signal intensity in T2-weighted imaging. The different signal intensity in the surrounding areas of lesions was considered an appearance of the edematous reaction. We described this edematous change as “acute on chronic phenomenon”. It meant that there was acute or subacute inflammatory reaction induced by crystal deposition. In this report, the “acute on chronic phenomenon” was found in eight patients whose duration of symptoms was less than 5 months. This unique imaging finding was different from that of the OLF. The images of OLF showed low-signal intensity without edematous change on both T1- and T2-weighted image. Other ten patients whose duration of symptoms more than 9 months did not have the imaging finding of “acute on chronic phenomenon”. However, we could not demonstrate a clear cut-off point for duration of symptoms to determine whether this phenomenon would be present or not.

CT scans were performed in five patients. The calcifications were seen as oval, high attenuation, and symmetric space-occupying lesions ventral to the lamina. They were located on either side of the midline and not observed in the capsular part. This condition could explain why most of patients presented with myelopathy instead of radiculopathy alone. For cervical spine calcifications, CT-scan frequently revealed characteristic “calcified railroad “pattern in the transverse retro-odontoid ligament, fine and mottled calcifications around the odontoid process, and intervertebral disc calcifications [26]. Unlike calcification, OLF would extent to the posterior facet joint because the initiation of the ossification starts at the joint capsule and the ligament was ossified unilaterally in some cases [27]. CPPD crystal deposits also result in enlarged retro-odontoid masses which should present with calcifications surrounding the apex of the dens. This condition is known as “crowned dens syndrome” [28] when patients present typical clinical features, including acute onset of severe neck pain, neck stiffness (particularly in rotation), and positive inflammatory indicators (fever, increased C-reactive protein level, or elevated white blood-cell count). However, calcification around the dens does not absolutely cause crowned dens syndrome. In addition, tumor and calcified retropharyngeal tendinitis may also have calcium deposition around the dens. Crown dens syndrome should be considered not only in CPPD disease but also in various clinical situations [29]. Salaffi et al. [30] reported that 51% of patients had periodontoid calcifications in known CPPD deposition in cervical ligamentum flavum. Haikal et al. [31] reported that 60% of patients had crowned dens syndrome compatible with their cervical computed tomography findings. They declared the importance of performing cervical computed tomography when evaluating patients with CPPD deposition could not be overemphasized. The incidence of the disease in our study was higher. By contrast, there is no typical retro-odontoid calcified lesion noted in patients with ossification of ligamentum flavum. A comparison between the CPPD deposition disease and ossification of the ligamentum flavum is summarized in Table 3 according to our study and reviewed literatures [10, 15, 23, 25, 27, 32].

**Table 3:** The distinctive imaging findings of CPPD deposition in ligamentum flavum and ossification of ligamentum flavum (OLF) in the cervical spine

	CPPD deposition	OLF
1. Morphology	Nodular	Beaklike or moundlike
2. Location	C4–5 and C5–6	No common location
3. Involvement of level	Multilevel predominant	Single level predominant
4. Side of the involved ligament	Always bilateral	Unilateral or bilateral
5. Extend to the posterior facet joints	Absent	Always present
6. Continuity with the lamina	No continuity	Continuity with lamina
7. Acute on chronic phenomenon	Depend on symptoms duration	Not reported
8. Retro-odontoid lesion	May present	Not reported

*CPPD* Calcium pyrophosphate dihydrate

The natural history of cervical myelopathy that resulted from CPPD deposition in the ligamentum flavum is not well-known. Prognostic factors include age, comorbidities, rate of deterioration, severity of symptoms, duration of symptoms, and signal change within the spinal cord [33]. It is generally agreed that surgical decompression is necessary for patients with ongoing and progressive symptoms refractory to conservative treatment [34]. However, for long-term effect of surgery, there is no significant difference between operative and non-operative group in a Cochrane Review of randomized controlled trials reported by Fouyas IP et al. [35] In patients with “acute on chronic phenomenon” in MR image, two to three grades of neurologic improvement according to Frankle scale were noted in our series. Two patients (cases 12 and 13) that suffered from severe and prolonged spinal cord compression with persisted and obvious signal change in MR image had shown one grade of improvement after treatment. The purpose of surgery was to prevent further decline in function in two patients. The options of surgical treatment in our case series include posterior decompression alone or decompressive surgery with posterolateral fusion. The long-term follow-up study showed that 91% of patients receiving laminectomy alone were satisfied with the outcomes and had significant improved functional status [36], but fusion with instrumentation still had its role in patients with significant neck pain, cervical lordosis less than 10 degrees, younger age, postoperative radiation, or the presence of instability [37]. For patients with neck pain, we did not routinely performed fusion surgery because neck pain could not always be considered as a predictor of late instability in elderly patients. We performed additional fusion surgery in a patient that demonstrated CPPD deposition in ligmentum flavum of C3–4 and C7-T1 to prevent instability in cervicothoracic junctional level.

This case series highlights important clinical issues as below. First, CPPD attacks of the cervical ligmentum flavum can result in neck pain, radiculopathy, myelopathy, or myeloradiculopathy. Second, nodular calcification of the ligmentum flavum in cervical spine raises high suspicion of CPPD deposition disease. Third, CPPD deposition can present with single or multilevel involvement. Fourth, CPPD deposition disease and ossification of ligamentum flavum are the different disease and have their own imaging features. We believe that the cases that suffered from CPPD deposition disease in the cervical ligmentum flavum is underreported because specimens are not routinely stained in Hematoxylin and Eosin staining or inspected with polarized-light microscopy. If CPPD deposition is suspected, the specimen should be fixed in 70% ethanol and separated from other sectioning bone or cartilaginous specimens [38]. Most importantly, the pathologist should be informed of the suspicion so that the specimen can be handled appropriately.

## Conclusions

On the basis of our study and literatures in these cases and the review of the literature, the imaging features and intraoperative findings of nodular calcifications in cervical ligamentum flavum raise highly suspicion for CPPD deposition. This disease is different from ossification of ligamentum flavum, and it can be recognized by specific image features. An accurate diagnosis can be established through histopathological examination of the specimen. Patients with neurological symptoms may generally require surgical decompression to improve the symptoms and prevent the progression.

## Data Availability

Data are available from Taipei Veterans General Hospital, Taiwan. The datasets used and/or analyzed during the current study are available from the corresponding author on reasonable request.

## Abbreviations

CPPD: Calcium pyrophosphate dihydrate
CT: computed tomography
MRI: magnetic resonance imaging
STIR: short tau inversion recovery
OLF: ossification of the ligamentum flavum

## References

[1] Zitnan D, Sitaj S (1963). Chondrocalcinosis articularis. Section I: clinical and radiological study. Ann Rheum Dis.

[2] Steinbach LS, Resnick D (2000). Calcium pyrophosphate dihydrate crystal deposition disease. Imaging perspectives. Curr Prol Diagn Radiol.

[3] Fye KH, Weinstein PR, Donald F (1999). Compressive cervical myelopathy due to calcium pyrophosphate dihydrate deposition disease. Arch Intern Med.

[4] Richards AJ, Hamilton EB (1976). Spinal changes in idiopathic chondrocalcinosis articularis. Rhuematol Rehabil.

[5] Chanchairujira K, Chung CB, Kim JY, Papakonstantinou O, Lee MH, Clopton P (2004). Intervertebral disk calcification of the spine in an elderly population: radiographic prevalence, location, and distribution and correlation with spinal degeneration. Radiology.

[6] Rosenthal AK, Ryan LM (2016). Calcium pyrophosphate deposition disease. N Engl J Med.

[7] Cipolletta E, Filippou G, Scirè CA, Di Matteo A, Di Battista J, Salaffi F (2021). The diagnostic value of conventional radiography and musculoskeletal ultrasonography in calcium pyrophosphate deposition disease: a systematic literature review and meta-analysis. Osteoarthr Cartil.

[8] Giulioni M, Zucchelli M, Damiani S (2007). Thoracic myelopathy caused by calcified ligamentum flavum. Joint Bone Spine.

[9] Frankel HL, Hancock DO, Hyslop G, Melzak J, Michaelis LS, Ungar GH (1969). The value of postural reduction in the initial management of closed injuries of the spine with paraplegia and tetraplegia. Paraplegia.

[10] Kawano N, Matsuhi T, Miyazawa S, Ilda H, Yada K, Kobayashi N (1988). Calcium pyrophosphate dihydrate crystal deposition disease in the cervical ligamentum flavum. J Neurosurg.

[11] Bada H, Maezawa Y, Kawahara N, Tomita K, Furuzawa N, Imura S (1993). Calcium crystal deposition in the ligamentum flavum of the cervical spine. Spine.

[12] Imai S, Hukuda S (1994). Cervical radiculopathy due to deposition of calcium pyrophosphate dehydrate crystals in the ligmentum flavum: historical and histological evaluation of attendant inflammation. J Spinal Disord.

[13] Iwasaki Y, Akino M, Abe H, Tsuru M, Tashiro K, Miyasaka K (1983). Calcification of the ligamentum flavum of the cervical spine. Report of four cases. J Neurosurg.

[14] Cabre P, Pascal-Moussellard H, Kaidomar S, Bucki B, Bardin T, Smadja D (2001). Six cases of cervical ligamentum flavum calcification in blacks in the French West Indies. Joint Bone Spine.

[15] Miyazawa N, Akiyama I (2007). Ossification of the ligamentum flavum of the cervical spine. J Neurosurg Sci.

[16] Hamilton EBD (1978). Disease associated with CPPD deposition disease. Arthritis Rheum.

[17] Kobayashi T, Miyakoshi N, Konno N, Abe E, Ishikawa Y, Shimada Y (2014). Acute neck pain caused by arthritis of the lateral atlantoaxial joint. Spine J.

[18] Kobayashi T, Miyakoshi N, Abe T (2016). Acute neck pain caused by pseudogout attack of calcified cervical yellow ligament: a case report. J Med Case Rep.

[19] Markiewitz A, Boumphrey F, Bauer T (1996). Calcium pyrophosphate dihydrate crystal deposition disease as a cause of lumbar canal stenosis. Spine.

[20] Nakajima K, Miyaoka M, Sumie H, Nakazato T, Ishii S (1984). Cervical radiculomeylopathy due to calcification of the ligamenta flava. Surg Neurol.

[21] Yoshida T, Sekijima Y, Hoshi K, Kaneko K, Hashimoto T (2002). Two patients with pseudogout manifested by severe neck pain. Rinsho Shinkeigaku.

[22] Lin S-H, Hsieh E-T, Wu T-Y, Chang C-W (2006). Cervical myelopathy induced by pseudogout in ligmentum flavum and retro-odontoid mass: a case report. Spinal Cord.

[23] Mousselard HP, Smadja D, Cabre P, Raynaud M, Catonne Y (1998). Ossification of the ligamentum flavum with severe myelopathy in a black patient. A case report. Spine.

[24] Sushil P, Anant K (1994). Ossified-calcified ligmentum flavum causing dorsal cord compression with computed tomography-magnetic resonance imaging features. Surg Neurol.

[25] Haraguchi K, Yamaki T, Kurokawa Y, Ohtaki M, Ibayashi Y, Uede T (1996). A case of calcification of the cervical ligamentum flavum. No Shinkei Geka.

[26] Moshrif A, Laredo JD, Bassiouni H, Abdelkareem M, Richette P, Rigon MR (2019). Spinal involvement with calcium pyrophosphate deposition disease in an academic rheumatology center: a series of 37 patients. Semin Arthritis Rheum.

[27] Miyasaka K, Kaneda K, Sato S (1983). Myelopathy due to ossification or calcification of the ligamentum flavum: radiologic and histologic evaluation. AJNR.

[28] Baysal T, Baysal O, Kutlu R, Karaman I, Mizrak B (2000). The crowned dens syndrome: a rare form of calcium pyrophosphate deposition disease. Eur Radiol.

[29] Awisat A, Rosner I, Rimar D, Rozenbaum M, Boulman N, Kaly L (2020). Crowned dens syndrome, yet another rheumatic disease imposter. Clin Rheumatol.

[30] Salaffi F, Carotti M, Guglielmi G, Passarini G, Grassi W (2008). The crowned dens syndrome as a cause of neck pain: clinical and computed tomography study in patients with calcium pyrophosphate dihydrate deposition disease. Clin Exp Rheumatol.

[31] Haikal A, Everist BM, Jetanalin P, Maz M (2020). Cervical CT-dependent diagnosis of crowned dens syndrome in calcium pyrophosphate Dihydrate crystal deposition disease. Am J Med.

[32] Inoue H, Seichi A, Kimura A, Endo T, Hoshino Y. Multiple-level ossification of the ligamentum flavum in the cervical spine combined with calcification of the cervical ligamentum flavum and posterior atlanto-axial membrane. Eur Spine J. 2013;22:416–20. 10.1007/s00586-012-2521-7 PMC3641269 23053758

[33] Shin JJ, Jin BH, Kim KS, Cho YE, Cho WH (2010). Intramedullary high signal intensity and neurological status as prognostic factors in cervical spondylotic myelopathy. Acta Neurochir.

[34] Lebl DR, Hughes A, Cammisa FP, O’Leary PF (2011). Cervical spondylotic myelopathy: pathophysiology, clinical presentation, and treatment. HSS J.

[35] Fouyas IP, Statham PF, Sandercock PA (2002). Cochrane review on the role of surgery in cervical spondylotic radiculomyelopathy. Spine (Phila Pa 1976).

[36] Aleixo Laiginhas AR, Silva PA, Pereira P, Vaz R (2015). Long-term clinical and radiological follow-up after laminectomy for cervical spondylotic myelopathy. Surg Neurol Int.

[37] McAllister BD, Rebholz BJ, Wang JC (2012). Is posterior fusion necessary with laminectomy in the cervical spine?. Surg Neurol Int.

[38] Zunkeler B, Schelper R, Menezes AH (1996). Periodontoid calcium pyrophosphate dehydrate deposition disease: “pseudogout” mass lesions of the craniocervical junction. J Neurosurg.

